# Keeping our children safe: piloting a hospital-based home-visitation program in Israel

**DOI:** 10.1186/s13584-022-00525-w

**Published:** 2022-04-11

**Authors:** Ligat Shalev, Anthony Luder, Sivan Spitzer, Danna Krupik, Jumanah Essa-Hadad, Mary C. J. Rudolf

**Affiliations:** 1 Department of Population Health, Azrieli Faculty of Medicine, Bar-Ilan University, POB 1589, Henrietta Szold 8, 1311502 Safed, Israel; 2 Ziv Medical Center, Safed, Israel

**Keywords:** Hospital-based intervention, Pre-school children, Home safety, Injury prevention, Home visit

## Abstract

**Background:**

Unintentional childhood injuries are a leading cause of morbidity and mortality worldwide. Attempts to prevent child home injuries have rarely been implemented in hospital settings which present an important opportunity for intervention. The SHABI (‘Keeping our Children Safe; SHomrim Al BetIchut Yeladenu’) program recruits at-risk families presenting with child injury to the Emergency Department. Medical/nursing students conduct two home visits and provide safety equipment and guidance. The objective of this study was to investigate the impact of SHABI on participating families’ home-safety.

**Methods:**

The pilot was conducted between May 2019 and March 2020 in northern Israel, an area with high child injury rates. Eligibility included families with preschool children who incurred a home injury. Home-safety was assessed by observation through the ‘Beterem’ checklist. Parents' views, knowledge, awareness of dangers and report of home injuries were assessed at the start of each visit.

**Results:**

352 of 773 eligible families agreed to be contacted. 135 participated, 98 completed both home visits. Significant improvement in home-safety items was observed 4 months after the first visit (14 [IQR12-16]) vs. (17 [IQR15-19]; *p* < 0.001), accompanied by an overall increase in home safety (Mean ± SD 71.9% ± 9.5% vs. 87.1% ± 8.6%; *p* < 0.001). 64% reported greater awareness of dangers, 60% affirmed home was safer, and 70% valued the equipment. No difference was found in the prevalence of injuries (14 of 98 families prior and 8 after the visit; *p* = 0.17). Home visitors reported benefiting from the experience of working with disadvantaged families.

**Conclusion:**

The program, which included recruitment in a hospital emergency setting and use of healthcare students as home visitors, was successfully implemented and accompanied by significant improvement in home safety with a non-significant trend of child injury decrease.

## Background

Unintentional injuries are one of the leading causes of childhood morbidity, disability and mortality worldwide [1–3]. It is the fifth leading cause of death for children under 4 years old in the USA [4] and the third in Israel [5]. Risk factors for child home injuries have been investigated and shown to relate to family characteristics such as ethnicity [6], low socio-economic status [7], and lack of parental supervision [8]. Child characteristics include boys [1], age less than four years [8], and disorders of hyperactivity [9]. In Israel, Arabs and ultra-Orthodox Jews are at higher risk for child injuries [10].

Various educational interventions for parents have been developed over the years. One well-studied approach is home-visitation, focusing on parental education for home safety practices and the use of safety devices [11]. Examination of the literature shows conflicting findings in terms of impact. In some cases, a significant reduction in child injuries was found [12, 13], while in others it was not [14, 15]. Likewise, some studies showed a significant improvement in home safety [16, 17], while others did not [18, 19]. There have been attempts to explore why results are discrepant [20–22], but no clear indicators for success have emerged. Outstanding questions remain such as the relative benefit of recruiting in the community or hospital settings, the optimal number of visits, and qualifications to conduct home visits.

Given the inevitable limitation of resources, it makes sense to target families most at risk for recurrent injuries [23]. One way to do so may be to base interventions in the hospital where children with more severe injuries are more likely to be captured, yet exploration of the literature shows that most interventions are community-based, delivered through primary care clinics or children's centers [24, 25]. Only six hospital-based interventions have been described [17, 19, 26–29]. Of these, four took a focus on preventing child injury [19, 26–28]; two used home-visitation guidance- one, the largest, contacted families some days after arrival at the hospital for an injury [28], and one contacted families from logs of a pediatric residency continuity clinic [26]; two recruited at the time of attendance but offered guidance in the Emergency Room alone [17, 27]. Only two studies focused on home safety and/or injury prevention as well as being designed as hospital-based interventions using home visits [26, 28]. Their results were inconclusive with no significant change in child injury rate [28], and either minor changes in home safety practices [28] or no change at all [26].

We developed and implemented a home-visitation intervention for families recruited in the Emergency Department at the time of presenting with a pre-school child for a non-intentional child injury. It was delivered through Ziv Medical Center in the Galilee, Israel’s northern periphery, a region that is home to diverse populations and has amongst the highest rates of child injury in the country, particularly in the ultra-Orthodox Jewish and Arab sectors [10]. We hoped that by targeting families at risk and high need for home safety education we could create a model that would be of value elsewhere. This paper is part of a larger study that assessed the implementation process of SHABI in the hospital setting using the Consolidated Framework for Implementation Science [30]. We describe here the intervention and evaluation of its pilot year examining the impact on home safety.

## Methods

### The SHABI (‘Keeping our Children Safe; SHomrim Al BetIchut Yeladenu’) program

SHABI aims to reduce the recurrence of child injuries by intervening with families who present to a hospital Emergency Department with a preschool child who experienced an unintentional injury at home. Home visits are conducted by trained home visitors within two weeks of the injury and again four months later. The visit involves a tour through the home with parents’ approval, and the installation of safety equipment. It includes cooking and eating areas, storage and use of detergents, pesticides and medicines, the bathroom, the electrical system, bedroom and living areas, balconies and stairs, rooftops and outdoor spaces. The safety of each area is checked, and any concerns and solutions are discussed, encouraging the parents to make suggestions for reducing hazards. A safety kit that includes a smoke detector, electric socket covers, locks for cupboards, and door stopper is provided, and help is offered to install the components. Two months later, the home visitor contacts the family by phone, offering further guidance and answering any questions. A second home visit is conducted four months after the first, and includes an additional tour of the house.

Following the outbreak of the COVID-19 pandemic in March 2020 and in accordance with Ministry of Health instructions, recruitment was suspended and the remaining second visits were conducted by telephone, rather than by a physical visit.

#### Training of home visitors

Eleven nursing and medical students, were trained to carry out home visits and were paid a modest stipend per visit. The training involved five sessions over eleven months, namely:

- Two sessions on the epidemiology of child injury, unintentional injury prevention, home-visiting practice, and relationship-building skills. The sessions were taught by a coach from ‘Beterem- Safe Kids Israel’- a non-profit organization that promotes child safety [31]
- A session on the Arab community with cultural competent guidance on how to adapt the visit, e.g. initial contact with the husband to get permission to arrange a visit with his wife. The session was led by two social workers, one the head of social services in a local Arab village
- A session on the ultra-Orthodox Jewish community with cultural competent guidance on how to adjust the visit to their needs, e.g. incorporating child injury stories as media exposure is restricted. The session was led by a local ultra-Orthodox Rabbi
- A session on conducting the second visit by telephone because of COVID-19 restrictions

## Setting

The pilot was delivered from May 2019 until March 2020, with the recruitment of families through the pediatric Emergency Department at Ziv Medical Center, which is located in the city of Safed in Israel’s northern periphery. The hospital's catchment area includes 170,000 residents, of whom 10% are 0–4 years old, and 21% are non-Jewish [32]. Safed and surrounding towns and villages rank low in socio-economic status [32], with a high proportion of ultra-Orthodox [33] and Arab [32] residents. The hospital is characterized by higher admissions, mortality and attendance rates for unintentional child injury than elsewhere in Israel [10].

### Recruitment of families

The Emergency Department nursing team recruited families during their visits to the hospital with an injured child. All ten nurses were trained at a session that included an explanation of SHABI and its inclusion criteria, namely children aged 0–5 years presenting with a home injury; residence in the hospital’s catchment area; and competence in spoken Hebrew. The intention was to recruit 100 families within a year.

### Evaluation

The study evaluated home safety, families’ awareness of hazards, and both families’ and home visitors’ views regarding the visits’ usefulness. Outcome measures from the first and second visits were compared.

#### Home safety environment

The home was evaluated using a checklist developed by ‘Beterem’ [31], and based on evidence regarding injury prevention [34, 35]. It includes 30 items divided into different areas of the home (safe/unsafe), which the home visitors completed with the family during the tour. The questionnaire did not undergo a formal validation process, but has been extensively used by Beterem trained staff in various ethnic sectors as well as in disadvantaged families, and has high internal consistency (0.8 using the KR-20 exam) [36].

#### Awareness of child home hazards

Families' awareness of dangers in the home was assessed through an open-ended question administered by the home visitor at the start of each visit. It included an open question adapted from Kendrick and colleagues (1999) [37], regarding their perceptions of significant dangers in the home and was chosen following review of published tools assessing awareness, as well as personal contact with researchers in the field.

#### Reported child injuries and families' views

Families' self-report on any injuries requiring medical care (primary or secondary) for their 0–5 year old children was examined through a questionnaire developed for this study as no relevant tool was found in the literature, and administered by the home visitor. Reporting was limited to four months before the first visit (excluding the index injury) and four months after the visit. Families' views regarding SHABI were evaluated through brief telephone interviews conducted after each visit by a researcher (LS). The interview included five questions, using a Likert scale of 1 (strongly disagree) to 5 (strongly agree). The questions assessed the contribution the safety equipment and the visit made to the safety of the home, new knowledge and awareness of home safety, as well as a yes/no question regarding recommending SHABI to friends. An open-ended question after the second visit asked about any difficulties in implementing the safety recommendations. Telephone interviews were recorded and transcribed.

#### Home visitors' views

Home visitors' views were evaluated through a questionnaire developed for this study, regarding their views on the usefulness of the visit to the family (1—strongly disagree, 5—strongly agree), and an open-ended question about their general views of the visit.

### Data analysis

Analysis of the checklist included data regarding safe and unsafe items per family, as well as relevant and irrelevant items (according to their presence in the house e.g. absence of a porch, or access to roof or items related to child age e.g. supervision during eating for children under four years, but irrelevant for a family with older children). Home safety percentage was calculated as the percentage of safe to relevant items per family. The change in home safety over 4 months was calculated by comparing home safety percentage at the second home visit with that found at the first home visit. Lastly home safety change was analyzed when data was collected through direct observation during the second home visit compared with data collected by telephone ‘visits’ due to COVID-19 restrictions.

Quantitative data was analyzed by SPSS (version 25.0). Descriptive statistics were used to describe the characteristics of the participants and the distribution of the content analysis on open-ended questions [38]. All normally distributed data were analyzed using unpaired or paired t-test. Data found to be non-normally distributed were analyzed using Mann–Whitney U test for independent subgroup, and Wilcoxon test for dependent subgroups. Comparisons of percentages between different groups were analyzed using *X*^2^. Close-ended questions using Likert scale responses were grouped as negative (1–2), neutral (3) or positive (4–5).

Qualitative analysis was conducted utilizing the theoretical and conceptual framework of CIFR (Consolidated Framework for Implementation Science [30]. The qualitative findings were categorized according to these constructs and explanatory content analysis [38].

## Results

### Recruitment (Fig. 1)

From May 2019 to March 2020, a total of 773 families met the program's eligibility criteria for participation, of whom 487 (63%) were approached by nurses; 352 agreed to be contacted by the service. 135 families agreed to participate and completed the first visit, and 98 the second visit. No significant sociodemographic differences were found between families approached and not approached in the Emergency Department, nor between families agreeing and refusing to participate.

**Fig. 1:**
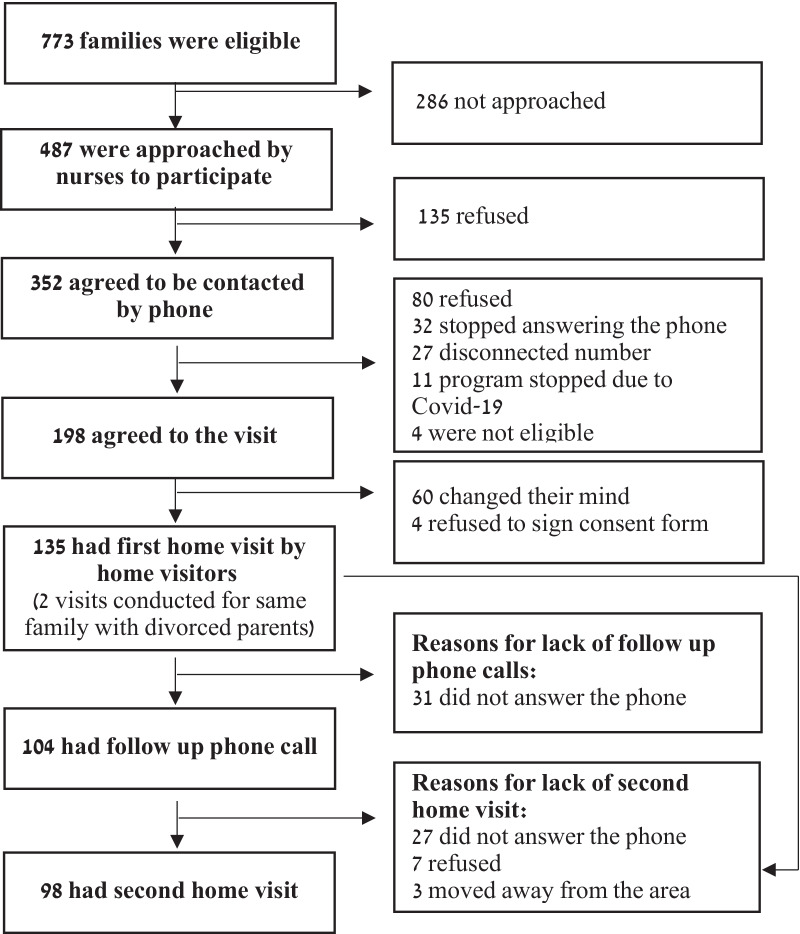
Flow chart of Emergency Department attendance for child injury and recruitment to SHABI

### Sociodemographic characteristics

Table 1 summarizes the socio-demographic characteristics of families who completed one visit and those who completed both visits. Fifty percent were ultra-Orthodox Jews and 11% Arab. A high proportion had < 12 years education and a third were unemployed. 28% of families had three or more children under the age of five. Only 6% of parents lived in separate households. Significantly less Arab families completed both visits and there was a trend for the less educated and unemployed to omit the second visit. No other significant differences were found between families who completed SHABI and families who had only one visit in terms of other sociodemographic characteristics and outcome measures.

**Table 1:** Sociodemographic characteristics of participating families in SHABI (n = 135)

	All participating families n = 135	Participating families		
		With second visit n = 98	Without second visit n = 37	*p* value
Sector				
Jewish^a^	120 (88.9%)	91 (92.9%)	29 (78.4%)	^b^*p* = 0.02
Arab	15 (11.1%)	7 (7.1%)	8 (21.6%)	
Jewish Ultra-orthodox	60 (50%)	45 (49.5%)	15 (51.7%)	^b^*p* = 0.69
Parents living in separate households	8 (5.9%)	4 (4.1%)	4 (10.8%)	^b^*p* = 0.16
Immigrants				
Mothers	11 (8.1%)	7 (7.1%)	4 (10.8%)	^b^*p* = 0.49
Fathers	15 (11.8%)	14 (14.9%)	1 (3%)	^b^*p* = 0.11
Age, mean (SD)				
Mothers	32.33 (5.88)	32.80 (5.47)	31.07 (6.77)	^c^*p* = 0.12
Fathers	35.39 (7.53)	35.99 (5.47)	33.73 (6.19)	^c^*p* = 0.13
≤ 12 years of education				
Mothers	45 (33.3%)	30 (30.6%)	15 (40.5%)	^b^*p* = 0.27
Fathers	55 (42.3%)	36 (37.9%)	19 (54.3%)	^b^*p* = 0.09
Unemployed				
Mothers	41 (30.6%)	27 (27.6%)	14 (38.9%)	^b^*p* = 0.20
Fathers	28 (21.7%)	24 (25.3%)	4 (11.8%)	^b^*p* = 0.10
# children in the family, mean (SD)	3.60 (1.98)	3.71 (2.08)	3.29 (1.71)	^c^*p* = 0.27
Families with > 3 children under 5 years old	38 (28.1%)	29 (29.6%)	9 (24.3%)	^b^*p* = 0.54

^a^'Jewish' included secular, traditional, religious, and ultra-Orthodox participants

^b^Comparison using Chi-squared test

^c^Comparison using t-test

### Home safety environment

**Fig. 2:**
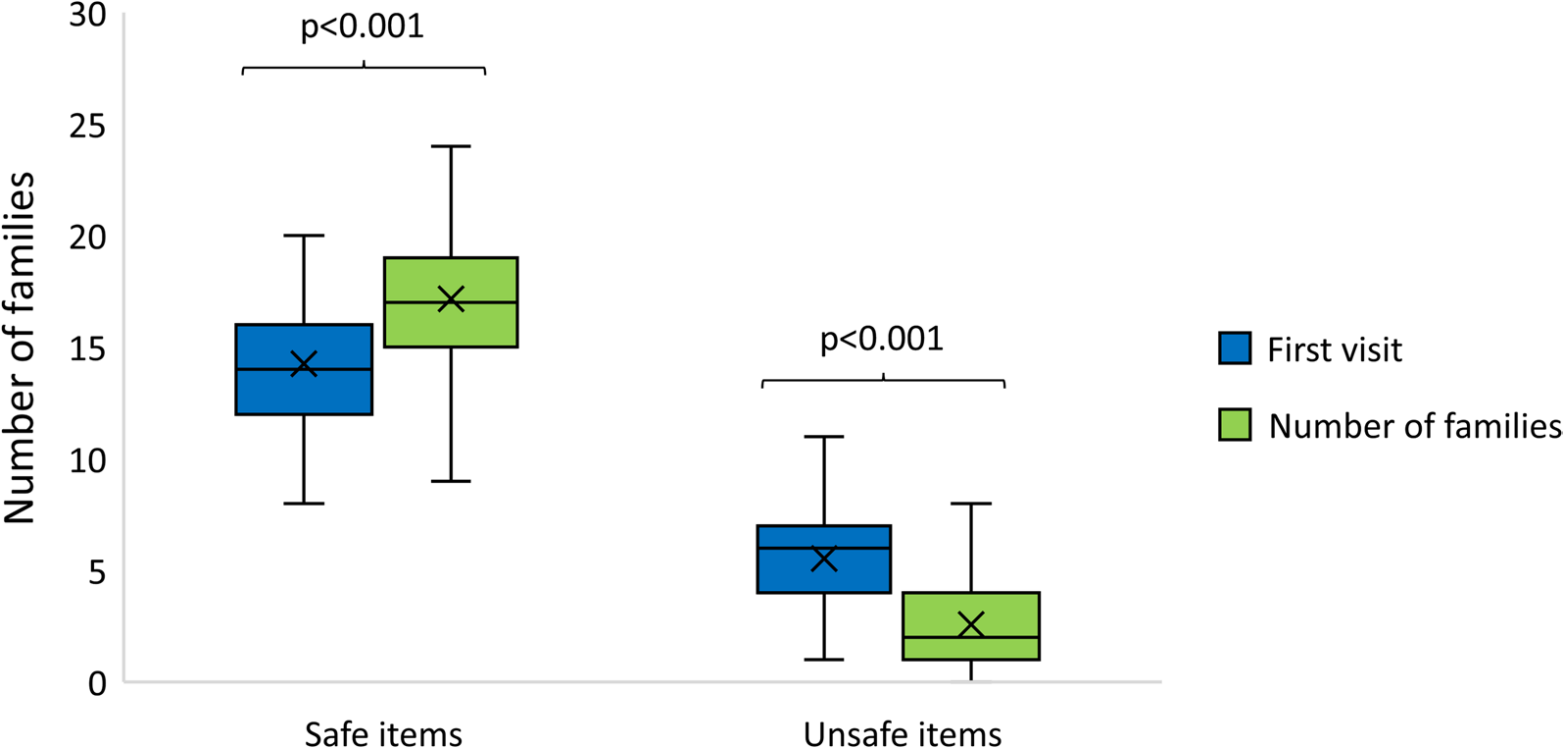
Median score of safe and unsafe checklist items in families who completed both visits (n = 98)

Figure 2 compares home safety assessed by the checklist at the first and second visit. A significant improvement was found in both safe items and unsafe items (Z = − 7.97, *p* < 0.001; Z = − 8.36, *p* < 0.001).

Individual checklist items were compared between the two visits (Table 2). Families appeared to readily adopt some behavioral recommendations such as keeping detergents and hot drinks out of children's reach, although were less likely to keep medicines out of reach or use back stove burners. Families also managed to adopt some structural recommendations such as installing safety equipment, but were less likely to install window bars and stair rails.

**Table 2:** Families' changes to the home and barriers they reported in implementing safety guidance (n = 98)

Recommendation^a^	No. of recommendations implemented			Parents’ comments regarding barriers to implementation^b^
	1st visit	2nd visit	*p* value^c^	
*Falls*				
**Anti-slamming devices installed on the doors**	**3 (3.1%)**	**62 (63.3%)**	***p* = 0.000**	Device perceived as low quality Useless because the doors are always open Not perceived as a danger
**Cabinets and bookcases fixed to the wall**	**28 (29.8%)**	**59 (62.1%)**	***p* = 0.000**	Time needed to make change Cannot fix alone- waiting for husband/ professional Not practical/possible Not perceived as a danger
Stair rails without bars or elements that allow climbing	18 (81.8%)	20 (100%)	*p* = 0.10	Too expensive
Furniture far from balcony railing	29 (74.4%)	31 (91.2%)	*p* = 0.06	
Porch rail without bars or elements that allow climbing	33 (84.6%)	34 (97.1%)	*p* = 0.11	
Gates at both ends of the stairs	4 (23.5%)	6 (40%)	*p* = 0.45	Too expensive Not practical/possible
Windows barred or have devices that limit opening	27 (48.2%)	32 (58.2%)	*p* = 0.29	Rental residence change Too expensive Time needed to make change
Rail surrounds the entire porch	37 (94.9%)	34 (97.1%)	*p* = 1.00	Too expensive
Safety rail on the child's bed	67 (98.5%)	66 (97.1%)	p = 1.00	
Rails down the length of staircases	22 (88%)	20 (90.9%)	*p* = 1.00	Too expensive
*Burns and scalds*				
**Smoke detector installed**	**22 (22.4%)**	**69 (70.4%)**	***p* = 0.000**	Rental residence Moving to new apartment soon and plan to install there Device perceived as low quality Not perceived as a danger
**Kettle or electric water pot close to the wall and out of child's reach**	**81 (88%)**	**90 (98.9%)**	***p* = 0.003**	
**Hot drinks served out of child's reach**	**80 (83.3%)**	**92 (94.8%)**	***p* = 0.01**	
Flammable materials kept high or locked	11 (55%)	15 (75%)	*p* = 0.18	
Cooking on gas burners at a distance from children	68 (70.1%)	78 (81.3%)	p = 0.07	Not practical/possible Hard and uncomfortable to cook only on far burners The burners for regular cooking are in front
Hot pots kept out of reach during cooking and mealtimes,	87 (88.8%)	92 (93.9%)	*p* = 0.20	Lack of out of reach areas due to small residence
*Electrocution*				
Electrical wires and sockets intact	89 (90.8%)	94 (95.9%)	*p* = 0.15	Cannot fix alone-waiting for husband/professional
Safety circuit breaker at home	97 (99%)	98 (100%)	*p* = 1.0	
*Poisoning*				
**Detergents kept in high or locked storage areas**	**48 (49%)**	**80 (81.6%)**	***p* = 0.000**	Lack of high storage area Accessibility Not perceived as a danger
**Pesticides kept in high or locked storage**	**53 (72.6%)**	**76 (96.2%)**	***p* = 0.000**	
Detergents and pesticides stored in their original packaging	95 (96.9%)	98 (100%)	*p* = 0.24	
Medicines and vitamins kept high or locked storage	88 (90.7%)	91 (92.9%)	*p* = 0.58	Not perceived as a danger Accessibility "I know it is dangerous but need it accessible and cannot put it away"
*Drowning*				
Paddling /plastic pools drained after use; large pools covered and fenced	60 (95.2%)	69 (100%)	*p* = 0.10	Not practical/possible
Tubs or bathtubs emptied after use	84 (97.7%)	83 (96.5%)	*p* = 1.0	
*Chocking/suffocation*				
**No playing with balloons/ plastic bags**	**49 (53.3%)**	**68 (73.9%)**	***p* = 0.004**	
Adult supervision while children eat	76 (88.4%)	80 (92%)	*p* = 0.42	Not practical/possible Not perceived as a danger "I know it is not safe, but it is not practical to supervise every time they eat" "I trust the kid's instincts not to choke"
*Car injury*				
Yard fence that separates parking area and children's play area	32 (72.7%)	36 (78.3%)	*p* = 0.54	Cannot fix alone- waiting for husband/ professional Rental residence Too expensive

^a^Significant differences between first and second visit indicated in bold

^b^All items regarding guard rails on the roof were excluded as irrelevant to this population

^c^Data was collected and analyzed through parent's telephone interviews, using Chi-squared test

Significant improvements in home safety were found in the category of falls, burns and scalds, poisoning, and choking/suffocation, but none for electrocution, drowning or car injury. In some, change was not possible due to high baseline safety rates. On telephone interviews, most parents saw the recommendations as necessary but found some difficult to apply due to time constraints; impracticality; requiring help from others; or cost. Other items were beyond families' control requiring landlords’ permission to make changes, or there was reluctance to invest in a house they did not own. A few recommendations were perceived as unimportant or not dangerous.

When expressed as percent safety of the household (excluding irrelevant items) there was a highly significant improvement (Mean ± SD 71.9% ± 9.5% vs. 87.1% ± 8.6%; t = -16.27, *p* < 0.001). No significant difference was found between home safety percentage delta improvement for families who completed the second visit via face-to-face contact (n = 55) and those who received a telephone call due to COVID-19 restrictions (n = 43) (Mean ± SD 14.9% ± 10.1% vs. 15.4% ± 8.1%; t = -0.99, *p* = 0.27).

### Awareness of child home hazards

Parents were asked at the start of each visit about the greatest dangers that children are exposed to in homes. Of the 98 families who completed both visits, there was strong awareness of falls (91 in the first visit vs. 79 in the second visit) and burns (54, 57), with few citing animal injuries (1, 2) or heatstroke in cars (0, 1). The number of categories reported by families at baseline was significantly higher than at follow up (Mean ± SDd 2.85 ± 1.19 vs. 2.11 ± 1.57; t = 5.25, *p* < 0.001).

### Families' views and reported injuries (Table 3)

Most parents reported high satisfaction with SHABI, its contribution to home safety and the safety equipment provided, after both visits. Only one third reported learning new knowledge at the first visit, and this reduced further at the second visit. By contrast, 64% reported raised awareness about home safety at the first visit, with a significant reduction to 44% at the second visit. After the first visit 122 (95%) of families said they would recommend SHABI to friends or family and this increased to 88 (98.9%), after the second visit.

**Table 3:** Families' views about the contribution of SHABI following the first and second visits (n = 135)

	Number of positive responses following all 135 first visits (%)	Number of positive responses in families completing both visits n = 98 (%)		
		After the first visit	After the second visit	*p* value^a^
The house is safer today for my child than before the home visit	62 (48.8%)	47 (50%)	52 (56.5%)	*p* = 0.11
The home visit helped to make my home safer	75 (57.7%)	57 (59.4%)	57 (62%)	*p* = 0.19
**The home visit provided new knowledge regarding home safety**	**41 (31.5%)**	**29 (30.2%)**	**15 (16.3%)**	***p* < 0.001**
**The home visit helped to raise my awareness towards home safety**	**80 (62.5%)**	**61 (64.2%)**	**39 (43.8%)**	***p* = 0.001**
The safety equipment helped to prevent injuries at home	96 (73.8%)	68 (70.8%)	65 (71.4%)	*p* = 0.41

^a^Comparison using Wilcoxon test

A significant change between the first visit compared to the second visit indicated in bold

Families reported on child injuries requiring medical care (primary or secondary) four months before participating in SHABI and four months later. Excluding the index injury, 14 of the families who completed both visits reported an injury prior to the first visit, while only eight reported an injury following the second visit. The difference was not significant (*p* = 0.17).

### Home visitors' views

Home visitors reported that they felt that 69% of the first visits and 74% of the second visits were useful or very useful. They also commented that the visits to ultra-Orthodox and Arab homes increased their understanding of cultural and religious groups with whom they had little familiarity.

## Discussion

This study describes the pilot of a hospital-based child injury intervention involving home visits and its impact on home safety. The findings indicate success in recruiting families in a hospital setting at the time of presenting with an injury, and positive results in terms of improvement in home safety and reported behavior change. Families affirmed that home safety was improved, their awareness of dangers enhanced and they appreciated the safety equipment. The study contributes to the field of injury prevention in demonstrating that recruitment of at risk families in the emergency setting is feasible, and that medical and nursing students can be trained to conduct home visits. The results suggest that improvement in home safety occurred four months following a single home visit, along with a non-significant decrease in the occurrence of child injuries.

Our hypothesis that families would be receptive to the idea of a home safety visit at the point where one of their children had incurred an injury was confirmed. Our target of 100 families, many of whom could be designated as high risk, was well exceeded. The feasibility of using a busy Emergency Department for this purpose is important as it may have relevance to other public health interventions, particularly in countries like Israel, where there is a limited interface between hospital and community [39, 40]. A literature review revealed only one hospital-based study that involved home visits following an injury, but recruitment of families occurred some days after attendance [28]. Another study recruited at the time of injury but guidance took place in the hospital and outcomes were based on families’ reports rather than observation [17].

The challenge for any public health interventions lies in achieving behavior change and not simply increasing knowledge [41]. This is evident in some child injury prevention trials, where knowledge about home safety increased but with only partial or no change in behavior [13, 16, 28, 37]. SHABI families, by contrast, reported a little gain in their knowledge levels, but more importantly did make improvements to the home which were observed four months following the first home visit.

The home visit checklist was core to the intervention. Its strengths lay in the way it created an educational guide for a visit, as well as a relatively objective way of assessing the home. Used sensitively, it provided a systematic but non-threatening structure to the visit, while allowing the collection of data. It also highlighted the difficulty of preventing child injury when living in rental accommodation, consistent with the qualitative findings by Smithson and colleagues (2010) in their systematic review [21]. The provision of safety equipment added to family participation, and was still appreciated four months after the first visit, although it is noteworthy that safety-equipment-based interventions have not been shown to help reduce injury rates [11, 42, 43].

The literature is unclear about the optimal number of visits required to improve home safety. Interventions range from two visits [28] to weekly visits over 18 months [18], with conflicting findings for home safety improvement and child injury reduction. Two trials utilizing a single visit failed to significantly improve home safety practices or recurrence of injuries [26, 28]. Indeed, although both families and home visitors felt they were of value, the added benefit of the second visit is unclear.

Our study utilized pre-clinical medical and nursing students as home visitors trained in child injury and cultural competence. Others have also employed non-professional home visitors [14, 15]. We found that the use of healthcare students had several advantages. Not only were costs kept to a minimum, but the experience exposed them to cultures they had not previously encountered in any real sense and helped change preconceptions. This may well impact on their cultural competence when working with disadvantaged populations.

There are limitations to our study. As a pilot study there were no controls, so no definitive conclusions can be made about effectiveness. Its primary outcome, home safety, was measured by observation through a checklist which also provided valuable structure to the intervention. However, it was completed by the home visitors so there was possible potential for bias. Unfortunately, sub-group analysis by ethnic group was not possible due to the small sample size for each individual group. It was reassuring that interviews conducted by an independent researcher confirmed parent's perceptions that home safety had increased. Lastly, change in injury rates, like in other studies, was calculated from family reports rather than medical records.

## Conclusions

SHABI proved to be a promising intervention. Recruiting in an emergency setting proved feasible, and showed that the traditional gap between hospital and community care could be bridged. We found that using health-profession students was successful. A larger sample size is required to demonstrate if the occurrence of injuries is decreased significantly and sustainably by this type of program. Ultimately a randomized controlled trial is needed to ascertain the extent of SHABI’s effectiveness.

## Data Availability

The datasets used and/or analyzed during the current study are available from the corresponding author on reasonable request.

## Abbreviation

SHABI: ‘Keeping our Children Safe; In Hebrew: SHomrim Al BetIchut Yeladenu
